# Time in Associative Learning: A Review on Temporal Maps

**DOI:** 10.3389/fnhum.2021.617943

**Published:** 2021-04-06

**Authors:** Midhula Chandran, Anna Thorwart

**Affiliations:** Department of Psychology, Philipps-Universität Marburg, Marburg, Germany

**Keywords:** associative learning, conditioning, time, temporal maps, temporal learning

## Abstract

Ability to recall the timing of events is a crucial aspect of associative learning. Yet, traditional theories of associative learning have often overlooked the role of time in learning association and shaping the behavioral outcome. They address temporal learning as an independent and parallel process. Temporal Coding Hypothesis is an attempt to bringing together the associative and non-associative aspects of learning. This account proposes temporal maps, a representation that encodes several aspects of a learned association, but attach considerable importance to the temporal aspect. A temporal map helps an agent to make inferences about missing information by applying an integration mechanism over a common element present in independently acquired temporal maps. We review the empirical evidence demonstrating the construct of temporal maps and discuss the importance of this concept in clinical and behavioral interventions.

## 1. Introduction

In a simple classical conditioning task, the computational goal of the agent is to accurately predict the arrival of the unconditioned stimulus (US) with respect to the conditioned stimulus (CS). To make an optimal conditioned response (CR), the agent must form an accurate representation of the order and timing of these two events. Suppose one often travels from train station A (CS1) to the airport (US). Assuming all other factors were ideal, the travel takes 30 min between the CS1 and the US. That is, the US always arrives 30 min after the CS1. After a several such trips, one would learn to predict the arrival of the airport, and hence could read a book for 30 min without paying attention to one's environment and only begin to prepare for the arrival (CR) in the last minute of this 30 min (of course they will be also other information available for predicting the arrival of the airport but they are not necessary). Here, the behavioral response (CR) is based not only on the learned statistical relationship between the two stimuli (that the airport would arrive after station A) but also on their precise temporal relationship (30 min).

Associative learning theories have evolved over the last 50 years and successfully accounted for several phenomena observed during a classical conditioning procedure (Thorwart and Livesey, 2016). Despite this success, they failed to account for several other aspects influencing the associative learning mechanism (Savastano and Miller, 1998; Delamater et al., 2014; Molet and Miller, 2014). An accumulating evidence suggests that associative learning is influenced by attentional (Le Pelley et al., 2016; Nobre and Van Ede, 2018), spatial (Blaisdell and Cook, 2005; Sawa et al., 2005), temporal (Honig, 1981; Cole et al., 1995; Barnet et al., 1997; Savastano and Miller, 1998; Leising et al., 2007), and qualitative (Holland, 1980, 1988; Blaisdell et al., 1997; Delamater, 2012) aspects as well. In this review, we are focusing on the encoding and representation of temporal aspects in associative learning. More specifically, we will examine the temporal maps construct, which is a mental representation encoding the temporal and associative aspects of an association (Savastano and Miller, 1998; Arcediano and Miller, 2002).

The earlier theories assumed that temporal and associative learning are parallel and independent processes. While temporal learning mechanisms were historically addressed from a perceptual point of view, associative learning theories focused on the statistical aspect of learning (Rescorla, 1972; Mackintosh, 1975; Pearce and Hall, 1980). The Temporal Coding Hypothesis (TCH) proposed by Miller et al. was a radical departure from this traditional approach and attempted to integrate the associative and temporal aspects of learning (Matzel et al., 1988; Barnet et al., 1997; Savastano and Miller, 1998; Arcediano and Miller, 2002). The four tenants of TCH are as follows:

(1) Temporal contiguity between events is both necessary and sufficient for the formation of the association.
(2) Temporal distribution of events in an association is encoded in the form of a temporal map (Honig, 1981).
(3) Temporal information plays an essential role in determining the topology, magnitude and timing of the conditioned response elicited when one of the associated events is presented.
(4) Subjects can superimpose independently acquired temporal maps, which share a common element and thereby allowing the expression of the temporal relationship between events that were never actually paired together.

## 2. Temporal Map

The highlight of TCH is the temporal map concept, which is an attempt to integrate the associative and temporal attributes of learning. A temporal map is a representation encoding the associative and the precise temporal parameters of an association (Savastano and Miller, 1998; Balsam and Gallistel, 2009). This representation helps the agent to make inferences about missing information by integrating independent temporal maps over common elements represented in them. When it comes to associative learning in humans, one can say that temporal features of events have a crucial influence on the behavior. To demonstrate this, let us go back to the previous example. Imagine one also often takes the train from station B (CS2) to the station A (CS1). Assuming all the other factors were ideal, the travel takes 60 min between CS2 and CS1. One could therefore acquire a temporal map, which encodes that CS1 would arrive 60 min after CS2. Since one already knows the temporal map of CS1 and the US from the previous experiences, one additionally could calculate the temporal distance between CS2 and the US by integrating the two temporal maps (CS2-CS1 and CS1-US). Thus, if one needs to travel for the first time from station B (CS2) to the airport, one is nevertheless able to predict that the airport (US) will arrive 90 min after the departure from station B (CS2) and can fall asleep for 89 min, and would not require the information about the arrival of station A (CS1) during the journey. Hence, one could start preparing for the arrival (CR) at the last minute of this 90 min. Such temporal integration could enable imagining possible future events through mental time traveling, which would be beyond the scope of the traditional associative learning theories.

**Figure 1:**
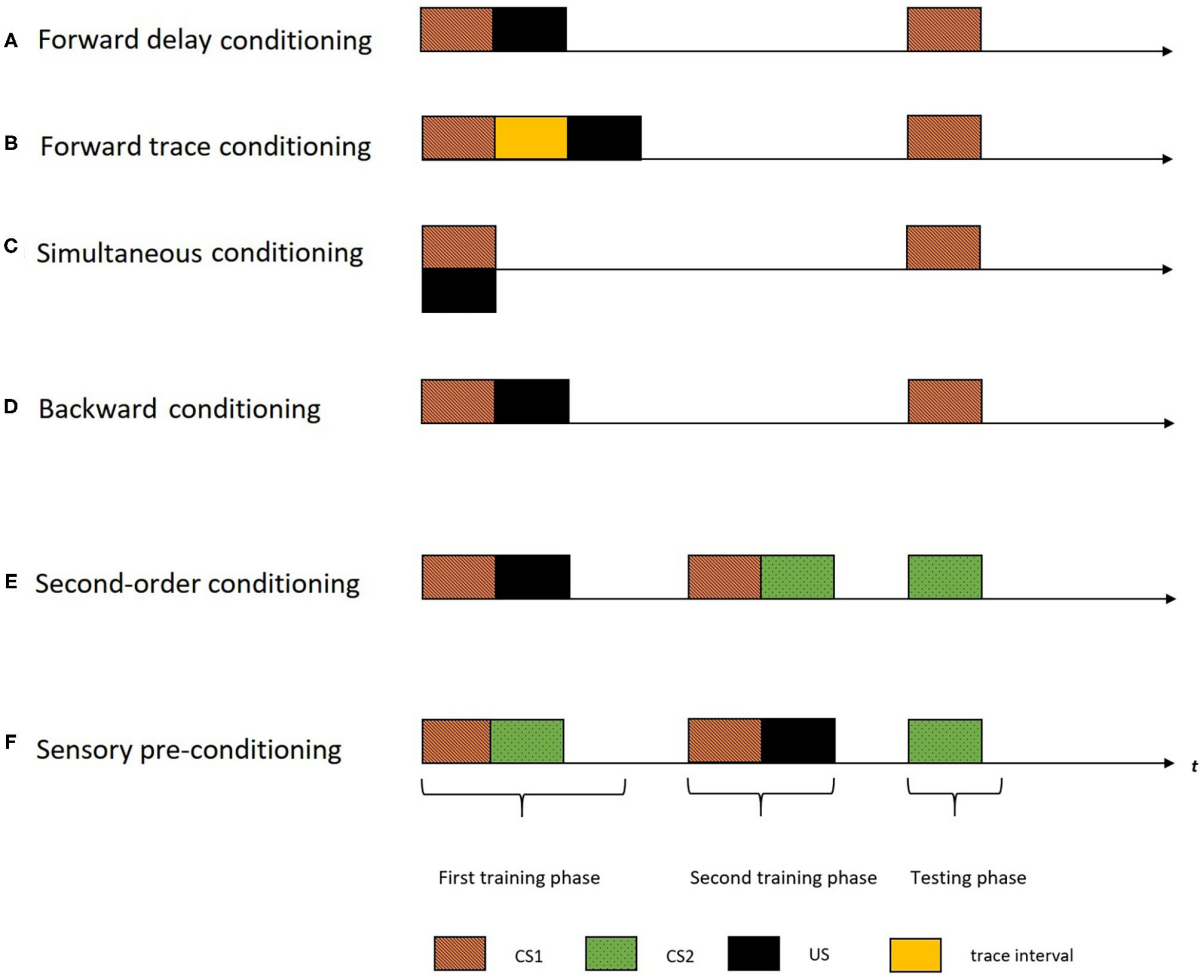
The figure indicates different temporal arrangements of events in a single trial of different conditioning procedures with the horizontal axis representing time (t). The black rectangles indicate the US, the colored rectangles with different patterns indicate different CS's, and the yellow rectangle indicate the trace interval between stimulus presentations. A standard first-order conditioning procedure involves two training phases followed by a testing phase. **(A)** In a forward delay arrangement, the US is presented right after the termination of CS1. **(B)** In a forward trace arrangement, the US is presented after a trace interval following the CS termination. **(C)** In a simultaneous arrangement, the CS and the US are presented at the same time. **(D)** In a backward arrangement, the CS is presented after the termination of the US. **(E)** A standard SOC involves two training phases followed by a testing phase. In the first training phase, CS1 and the US are presented (forming a CS1-US association). And, in the second training phase, the two CS's (CS1 and CS2) are presented (forming a CS1-CS2 association). During the testing phase, the second order stimulus (CS2) is presented. **(F)** The SPC procedure is similar to the SOC, only with a reversed order of the two training phases (CS1-CS2 pairing followed by CS2-US pairing). The testing phase involve presentation of CS2.

Although the concept of cognitive maps is popular since Tolman (1948) (i.e., spatial maps), many questions about these constructs remain yet unexplored, which is the case for the temporal maps as well. The empirical studies have adapted first-order (by manipulating the temporal distributions of the stimuli) and higher-order conditioning procedures [such as second-order conditioning (SOC) and sensory pre-conditioning procedures (SPC)] to explore the construct of temporal maps in humans and animals. Figure 1 demonstrates the different temporal distribution of events used in the first-order conditioning procedures. There are mainly four variants of temporal arrangements in a first-order conditioning procedure: forward delay, forward trace, simultaneous and backward arrangement. In a forward delay arrangement, the US is presented right after the termination of CS1 (Figure 1A). In a forward trace arrangement, the US is presented after a trace interval following the CS1 termination (Figure 1B). In a simultaneous arrangement, the CS1 and US are presented at the same time (Figure 1C). In a backward arrangement, the CS1 is presented after the termination of the US (Figure 1D). A standard SOC procedure (Figure 1E), involves two training phases followed by a testing phase. In the first training phase, CS1 and US is presented (forming a CS1-US association). In the second training phase that followed, two CS's (CS1 and CS2) are presented (forming a CS1-CS2 association). This training (CS1-US and CS1-CS2) would result in the formation of a novel association of CS2 and the US. Hence, in the testing phase, despite not having a prior association with the US, CS2 comes to elicit a CR. The SPC procedure (Figure 1F) is similar to SOC, only with a reversed order of the two training phases (CS1-CS2 pairing followed by CS2-US pairing). In the rest of this review, we will use these terminologies while inspecting the empirical evidence demonstrating the temporal maps construct.

**Figure 2:**
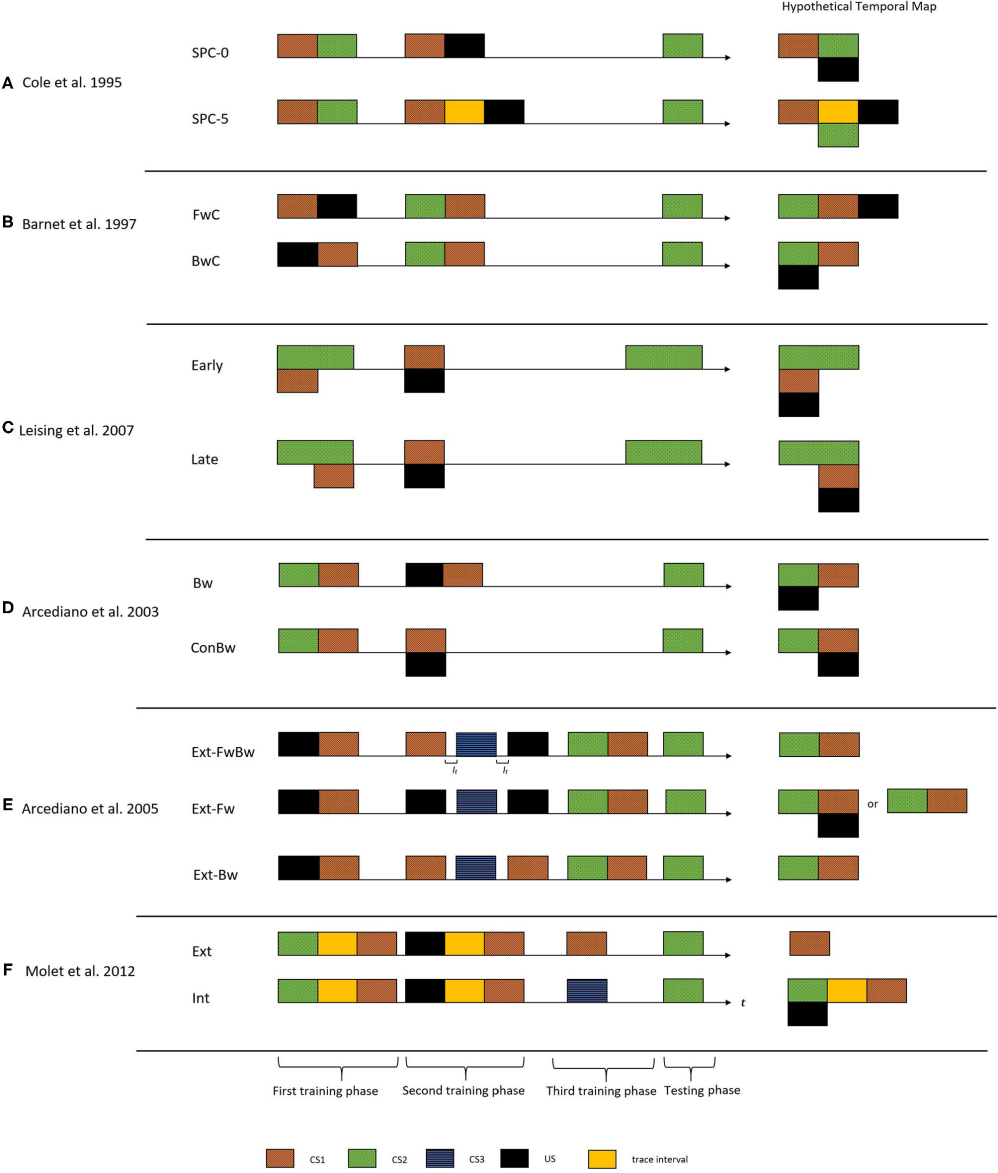
The figure presents the critical experimental groups of various studies exploring the temporal maps construct. On the left side is the treatment administered to the different groups. The horizontal axis indicate the time (t) and I_*t*_ indicate the inter-trial interval. The black rectangle indicates the US, the colored rectangles with patterns indicate different CS's, and the yellow rectangle indicates the trace interval between stimulus presentations. On the right side is the corresponding hypothetical temporal maps formed after integration process for each of these groups. These temporal maps predict the CR from the TCH point of view upon presentation of CS2 in the testing phase. That is the CR observed would be timed based on the CS2-US relationship depicted in the integrated temporal map. **(A)** Cole et al. (1995) demonstrated temporal maps encoding associations with trace and delay arrangement using the SPC procedure. **(B)** Barnet et al. (1997) used a SOC procedure to demonstrate that animals can encode backward associations in temporal maps. **(C)** Leising et al. (2007) used a more systematic approach to demonstrate that the temporal maps encode the specific time intervals and duration of events. **(D)** Arcediano et al. (2003) demonstrated humans encoding backward associations in temporal maps. **(E)** Arcediano et al. (2005) examined the directional nature of temporal maps in humans by administering a modified version of SOC procedure. **(F)** Molet et al. (2012) demonstrated that animals apply the integration mechanism to independent temporal maps at the time of testing.

Cole et al. (1995) made one of the earliest demonstrations of encoding trace and delay arrangement relationships in temporal maps. Figure 2A shows the critical experimental groups of this study. They administered a temporally manipulated SPC procedure on two groups of rats (Group SPC-0 and Group SPC-5). Both groups received the same training during the first phase (CS1-CS2). In the second phase, Group SPC-0 received a delay arrangement of CS1-US pair, whereas Group SPC-5 received a trace arrangement (5-s trace interval). The right side of Figure 2A demonstrates the resultant hypothetical temporal maps from the integration process. According to this proposition, Group SOC-0 would form a temporal map depicting an association with a simultaneous arrangement of CS2 and US (hence a weak CR), whereas for Group SOC-5, it would be an association with a delay arrangement. The results supported these predictions and indicated a stronger CR to CS2 in Group SOC-5 compared to Group SOC-0.

According to most traditional learning theories, an agent learns an association only if it can provide information to predict the US. For example, they suggest that animals fail to learn associations in a backward or simultaneous arrangement. The weak CR observed toward the CS's following simultaneous or backward association learning procedures supported this claim (Pavlov, 1927; Cooper, 1991). From a computational point of view, it may seem plausible that an agent with limited cognitive capacity learns only about information that is crucial for their survival. However, temporal map studies have indicated that animals learn associations irrespective of their predictive aspects and that the empirical evidence demonstrating weak conditioning is simply a failure in capturing the learned association. In other words, TCH draws a critical distinction between learning an association and expression of this learning. This in turn challenges the frequently voiced position that associative learning depends on there being a prediction error (e.g., Rescorla, 1972) as the need for a “prediction” error *per se* is refuted by the demonstrations of simultaneous conditioning, and particularly backward conditioning, that are the empirical foundation of TCH.

A few studies examined such a performance deficit account of simultaneous and backward variants of higher-order conditioning procedures (Barnet et al., 1991, 1997; Arcediano et al., 2003). Using the SPC procedure in two groups of rats, Barnet et al. (1997) demonstrated that animals could learn backward associations and encode it in the temporal maps. The first group (group FwC) received a forward-delay conditioning arrangement of CS1-US and the second group (group BwC) received a backward delay conditioning arrangement of US-CS1 in the first training phase. Both groups received a forward pairing of CS2-CS1 in the second training phase. Upon examining the CR toward CS2, group BwC showed stronger CR compared to group FwC. According to the predictions of the temporal maps, the integration mechanism would result in CS2 and US to form an association with a simultaneous arrangement for group BwC, hence producing a stronger CR during the testing. For group BwC, an association with a trace arrangement between CS2 and the US would result in a weaker CR during the testing (see the hypothetical temporal maps indicted in Figure 2B). Their results were in line with these predictions and supported the claim that the relatively weak CR observed during simultaneous conditioning could be attributed as a performance deficit. However, both Cole et al. (1995) and Barnet et al. (1997) used only the magnitude of the CR as a measure of the integration mechanism.

More systematic evidence on encoding the specific temporal interval and duration from the observed CR came from Leising et al. (2007) (in rats) and Arcediano et al. (2003) (in humans and rats). Leising et al. (2007) used the SPC procedure to demonstrate a specific temporal expectation of the US using two experimental groups (Group Early and Group Late). During the first training phase, the animals received a group-specific manipulation of CS2-CS1 compound (Figure 2C). For Group Early, CS1 was presented during the early portion of CS2, whereas for Group Late, CS1 was in the latter portion of CS2. During phase 2, both groups received a presentation of the CS1-US pair in a simultaneous arrangement. From a temporal integration perspective, if the resultant temporal map encodes specific temporal intervals, Group Early would show a higher rate of CR during the early portions of CS2. But for Group Late, it would be during the latter portions of CS2 (as the hypothetical temporal maps indicted in Figure 2C). The temporal pattern of CR supported the idea that temporal maps encode specific time intervals of an association.

One could say that humans use temporal maps to make inferences during situations with incomplete information, as we discussed in the level crossing scenario. However, we came across relatively fewer studies exploring this construct in humans. Arcediano et al. (2003) is one of those few studies that examined this construct in human associative learning. They administered the SPC procedure in two experimental groups (Group Bw and Group ConBw). During the first training phase, both groups received CS2-CS1 pairings in a delay arrangement. During the second training phase, Group Bw received a backward arrangement of US-CS1 and Group ConBw received simultaneous arrangement of CS1 and US. The authors predicted that the temporal integration mechanism would result in CS2 and the US forming an association in a forward delay arrangement in Group ConBw and a simultaneous arrangement in Group Bw (see the hypothetical temporal maps indicated in Figure 2D). These results demonstrated that temporal maps could encode temporal intervals irrespective of the directional nature of the association. Also, it supported the idea that the weaker CR observed during a backward conditioning could be attributed to a performance deficit rather than a learning deficit.

### 2.1. Integrating Temporal Maps

Several studies followed up to investigate the structural properties of temporal maps (Arcediano et al., 2005; Polack et al., 2013; Craddock et al., 2018). Arcediano et al. (2005) examined the directional nature of temporal maps in humans (experiment 1) and non-humans (experiment 2A–2C). They examined two possible directional structures for a temporal map. It could involve a single bidirectional association or two independent unidirectional associations. Arcediano et al. (2005) administered a modified SOC procedure in three groups of participants. This procedure involved three training phases followed by a testing phase. As shown in Figure 2E, all three groups (Group Ext-FwBw, Group Ext-Fw, and Group Ext-Bw) received a backward delay arrangement of CS1 and US during the first training phase. The second training phase involved a direction-specific extinction treatment of the integration mediating element (see the hypothetical temporal maps expected for the three groups in Figure 2E). For group Ext-FwBw, three trials in this phase involved presentation of CS1, CS3, and US presented alone. Hence, these trials extinguished both forward (US→CS1) and backward (US←CS1) associations (from bi-directional point of view). For group Ext-Fw, only the forward association (US→CS1) was extinguished as they received presentation of CS3 (once) and the US (twice). For group Ext-Bw, only backward association (US←CS1) was extinguished as they received presentation of CS1 (twice) and CS3 (once).

According to the predictions of the bidirectional account, the second training phase would result in an extinction of the CS1- US association. Hence, the participant would encode only CS2-CS1 association from the training phase. As for all the three groups, the CS1-US association was extinct, the participants would fail to form the bidirectional link. Hence, during the testing phase, they would produce little conditioned response. The unidirectional account predicted group specific CR pattern during the testing. For group Ext-FwBw, the second training phase would extinguish the CS1-US association in both directions (through the CS1 and US trials). Hence the experimenter predicted little CR during the testing phase. For group Ext-Bw, the extinction treatment would extinguish only the US←CS1 association with the US trial. Hence, the unidirectional account predicted again a weak CR during the testing phase. In contrast, a strong conditioned responding to CS2 was expected from group Ext-Fw, as the extinction treatment only extinguished the US→CS1 (forward) association.

Let us refer back to the earlier example, where you learned the association between CS2 and the US. If you happen to travel from the US back to CS2, you would make the same prediction regarding the temporal distance between US and CS2. That is, assuming that all other factors were ideal, it will take 90 min to arrive at CS2 from the US. Implying that representing the temporal information not only influence behavior by making predictions about future events but also enables mental time traveling to imagine events that happened in the past. However, this conclusion seems a bit premature as another line of studies had suggested formation of an unidirectional link under certain circumstances (see Iliescu et al., 2020 for a more discussion on this directional nature of the association using the HeiDi model). This would bring us to the question that when does an agent applies this integration mechanism on the independent temporal maps. Either an agent integrates the independent temporal maps during the learning process (second training phase) or according to the demands of the situation (testing phase).

Suppose one assumes that the integration takes place during the (second) training phase (let us refer to the Group SPC-0 of Cole et al., 1995). In that case, it could be the result of an indirect association formed between the activated representation of CS2 and the US present in the trial. On the other hand, if one assumes that the integration process takes place during the testing phase, it implies that an agent stores the temporal maps as independent representations and perform the integration operation according to the requirement of the situation. Earlier studies could not account for this question, as their results are interpretable in either direction. Molet et al. (2012) and Polack et al. (2013) explored this further using the higher-order conditioning procedures in rats.

They used an SPC procedure in two experimental groups of rats (Group Ext and Group Int). The two groups received the same SPC procedure followed by a group-specific extinction phase (see Figure 2F). Group Ext received an operational extinction of CS2, whereas Group Int received a presentation of a novel stimulus (CS3). If the integration operation was applied during the learning phase, the extinction of the mediating element following the learning phase would not influence the integration mechanism. Whereas, if the integration operation was applied during the testing phase, this would result in the failure of the integration mechanism and thereby weak CR. The results were in line with the latter prediction, which led (Molet et al., 2012) to make an amendment to the fourth tenant of TCH to: “*subjects can superimpose temporal relations at the time of testing when they share a common element, thereby allowing for the expression at the time of testing of temporal relationships between cues that were never actually paired*” (Molet et al., 2012). Furthermore, few studies using rats (Molet et al., 2010b), pigeons (Cheng et al., 1996), and humans (Molet et al., 2010a) also indicted that integration of spatial associations with common information could be also addressed with the TCH framework.

## 3. Conclusion and Future Directions

The main takeaway is that humans and animals form temporal maps during the earliest of the learning phase, much before this learning influences its behavioral expression. This indicate the sensitivity to the temporal aspects in associative learning. The current review focused on excitatory learning, but temporal aspects will also affect for example inhibitory learning (see the review Molet and Miller, 2014). However, we want to also point out that the construct of temporal maps is rather new, and unexplored in many aspects. Miller et al. defined temporal maps as “*a representation of the total sum of all the encoded relationships between two events, with temporal information being of considerable importance, but not the sole content of the association*” (Arcediano and Miller, 2002). Implying, other non-associative aspects could also be encoded in a temporal map. Future studies guided by this view should examine these unexplored dimensions of temporal maps in humans. Especially as when it comes to human associative learning, time could be a crucial factor guiding many aspects of behavior. Understanding the nature of information encoded in an association has crucial implications in clinical domain.

Associative learning is a core mechanism of most mental disorders where a traumatic experience (US) is associated with a neutral event (CS) (Rief et al., 2015). The existing clinical interventions to treat these disorders are designed to break patients' expectations that are based on their situation-specific associations. A crucial question in this domain is that why is it difficult for patients to alter a learned expectation, for example by learning additional associations, when making new experiences, which contradict the expectation. Craske et al. (2014) suggested that introducing maximal mismatch with the learned expectation could be a key to more effective clinical interventions. The idea that the temporal information forms a crucial aspect of an association brings a new possibility to optimize these interventions further. As associative learning process involves encoding the temporal aspects at the earliest, it could be possible that they are crucial for the new learning process as well. Most of the interventions used in psychotherapy are trial-based approaches focused on breaking the prediction of “whether” an event is expected, not “when” an event is expected. We would like to conclude with the suggestion that future studies should further investigate this idea and examine if this approach can bring more effective outcome.

## Author Contributions

MC was responsible for the conception, drafting, and revising of the paper with support from AT.

## Conflict of Interest

The authors declare that the research was conducted in the absence of any commercial or financial relationships that could be construed as a potential conflict of interest.
